# Recent Progress in Carbon Nanotube Polymer Composites in Tissue Engineering and Regeneration

**DOI:** 10.3390/ijms21176440

**Published:** 2020-09-03

**Authors:** Gangadhar Lekshmi, Siva Sankar Sana, Van-Huy Nguyen, Thi Hong Chuong Nguyen, Chinh Chien Nguyen, Quyet Van Le, Wanxi Peng

**Affiliations:** 1 Department of Nanotechnology, Noorul Islam Centre for Higher Education, Kumaracoil, Thucklay, Kanyakumari, Tamilnadu 629180, India; lekshmigangadhar.nano@gmail.com; 2 Department of Material Science and Nanotechnology, Yogivemana University, Kadapa 516005, India; 3 Department for Management of Science and Technology Development, Ton Duc Thang University, Ho Chi Minh City 700000, Vietnam; nguyenvanhuy@tdtu.edu.vn; 4 Faculty of Applied Sciences, Ton Duc Thang University, Ho Chi Minh City 700000, Vietnam; 5 Institute of Research and Development, Duy Tan University, Da Nang 550000, Vietnam; nguyenthongchuong@duytan.edu.vn (T.H.C.N.); nguyenchinhchien@duytan.edu.vn (C.C.N.); 6 Faculty of Environmental and Chemical Engineering, Duy Tan University, Da Nang 550000, Vietnam; 7 Henan Province Engineering Research Center for Biomass Value-added Products, School of Forestry, Henan Agricultural University, Zhengzhou 450002, China

**Keywords:** CNTs, nanoparticles, tissue engineering, drug delivery, regenerative medicine

## Abstract

Scaffolds are important to tissue regeneration and engineering because they can sustain the continuous release of various cell types and provide a location where new bone-forming cells can attach and propagate. Scaffolds produced from diverse processes have been studied and analyzed in recent decades. They are structurally efficient for improving cell affinity and synthetic and mechanical strength. Carbon nanotubes are spongy nanoparticles with high strength and thermal inertness, and they have been used as filler particles in the manufacturing industry to increase the performance of scaffold particles. The regeneration of tissue and organs requires a significant level of spatial and temporal control over physiological processes, as well as experiments in actual environments. This has led to an upsurge in the use of nanoparticle-based tissue scaffolds with numerous cell types for contrast imaging and managing scaffold characteristics. In this review, we emphasize the usage of carbon nanotubes (CNTs) and CNT–polymer composites in tissue engineering and regenerative medicine and also summarize challenges and prospects for their potential applications in different areas.

## 1. Introduction

Rapid developments in nanotechnology (NT) and nanomechanical engineering should allow the use of more efficient production processes with lower energy usage and fewer negative environmental impacts. The latest research developments have involved the design and development of tissue engineering and regenerative medicine solutions. Carbon-based nanotubes, liposomes, and dendrimers are major examples of nanomaterials (NMs) intended for medicinal use. NMs can be either raw materials, intermediates, or mixtures of processed substances and unprocessed molecules, in which 50% of the molecules have diameters varying from 1 to 100 nm. NMs are used in surgeries and preventive medicine. For example, nanobeams are employed as parts of immune sensors or for stabilizing polymer composites. The small size of these materials gives rise to their material strength and functionality, but it also leads to significant concerns [1,2,3].

Nanoparticles (NPs) provide a higher degree of control over scaffold attributes, such as the capability to tune their mechanical strength and manage the release of active agents [4,5,6,7,8]. Significant disadvantages include a lower solubility, unpredictable biological activity, and shorter lifespans of biologically active compounds for cell development, such as antagonists and genetic materials [4,5,6,7,8]. The fabrication of NMs involves the synthesis of NPs that are extensively utilized for a broad range of products. It is essential to form NPs with sizes that vary from 10 to 1000 nm and are stable as colloids [9]. Particle formulations have certain benefits, such as extremely high surface potential and large specific surface areas with adjustable particle sizes, which makes them popular for use in tissue engineering and regenerative medicine for scanning, mechanical property improvement, biological ink additives, and antibacterial and biological products [8,10].

Furthermore, allograft bone grafting can only provide substrates due to cell loss and modified growth factors. Synthetic bone contains hydroxyapatite, collagen, and a composite resin scaffold [11]; nevertheless, there are only a few materials that can be used for synthetic bones. Scaffold processing is important for regenerative engineering, and there is a growing body of scientific literature on the use of carbon nanotubes (CNTs) as substrates [12]. In jaw regenerative medicine, wet lab research in 2002 showed that a polylactic acid–CNT composite enhanced the propagation of osteoblast cells [10,13], and subsequent studies showed that polycarbonate–urethane composites improved the adhesion of osteoblast cells [14,15]. Tissue engineering scaffolds require a material in which cells can multiply, enhance variation by cell growth features, and sustain mechanical strength to produce better outcomes than autografts. Numerous lab-based studies have displayed their specific functions on jaw-related cells [16].

The aim of this review to explain the importance of CNTs in bone tissue engineering and regeneration. Recent advances in CNTs and CNT-based composites that have investigated bone scaffolds or strengthening agents are discussed. Next, the successes of CNT-based composites for tissue engineering and regeneration are summarized and discussed. The remaining challenges are highlighted, and future directions for the growth of CNTs and their composites for tissue engineering and regeneration are provided.

## 2. CNTs in Tissue Engineering and Regenerative Medicine

### 2.1. CNTs in Tissue Engineering

**Figure 1:**
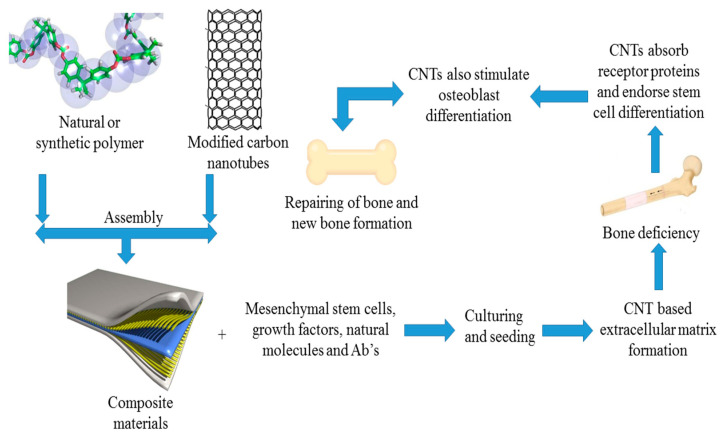
Schematic representing carbon nanotubes (CNTs) as a nanocomposite-based scaffold for use in bone regeneration and tissue engineering.

CNTs are carbon allotropes that comprise carbon molecules exclusively bound to one another by sp^2^ bonds [17]. CNTs may be viewed as one sheet of graphene wrapped into a cylindrical nanoparticle. They are typically subdivided into single-walled and multiwalled carbon nanotubes (MWCNTs). Single-walled CNTs comprise several dense tubular graphene sheets, and MWCNTs comprise various concentric tubular sheets. Generally, single-walled CNTs display a tightly packed hexagonal array around 1 nanometer in diameter and more than 1 mm in thickness. MWCNTs have a structure identical to porous graphite fibers and a larger diameter than single-walled CNTs, of between 2 and 100 nm [18]. On a cellular structure basis, CNTs in composites can be made from biocompatible nanocrystals (NCs) of collagen fibrils for the reconstruction and engineering of bone cells. These may enhance effective cellular communication with enzyme-binding proteins [19,20,21,22] and control cell physiology and increase stem cell distinction due to their favorable high cell constants; osteogenic differentiation and apatite mineralization stimulation to facilitate bone regeneration are depicted in Figure 1.

Scientists have shown that MWCNTs can oxidize and aggregate enzymes such as rhBMP-2, stimulating the activation of alkaline phosphatase and genomes Cbfa1 along with COLIA1, which then encourages osteogenic discrepancy of cultured cells of mesenchymal stem cells distinguished from human adipose. Additionally, MWCNTs often stimulate in vivo ectopic bone regeneration in mice dorsal muscles, indicating their ability to control downstream gene therapy reactions without adding exogenous signaling molecules or other specific ligands. Thus, this CNT material is also conducive to renewable bone tissue cultivation. The axial strength, resistance, and modulus of elasticity of natural CNT scaffolds are much greater than those of bone cells that are not correctly connected to body cells.

Carbon nanotubes can only perform their distinctive mechanical, electrical, and surface characteristics via structural integration with the other components, which then increases the overall physicochemical properties of the composites and joint viscosity [23]. Figure 1 outlines the uses of CNTs in tissue engineering and regeneration.

### 2.2. CNTs in Regenerative Medicine

**Figure 2:**
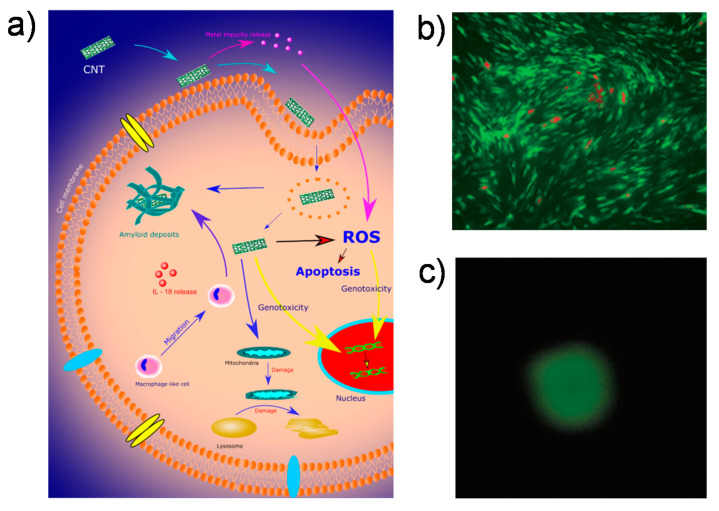
(**a**) Several cellular responses to CNT-induced toxicity. Adapted with permission from [17]; (**b**) live/dead cell assay of a polyethylene glycol–multiwalled CNT (PEG–MWCNT) composite (dead cells displayed in red); (**c**) the comet assay of PEG–MWCNT. Adapted with permission from [47].

CNTs have strong functional and morphological features and are of great importance for bone implants and design regarding biomaterials (BMs); therefore, the therapeutic use of CNTs for managing orthopedic disorders also faces many obstacles. Currently, the toxicity and porosity of CNTs are the most important problems restricting their use. The toxicity of CNTs is demonstrated in Figure 2a [17].

CNTs are hydrophobic due to nonpolar covalent bonds and lipophilicity [24,25,26,27,28]. Such enhanced surface connections, supported through van der Waals interactions, along with rod-designed frameworks, generally form CNT aggregates, which can significantly reduce the mechanical and electrothermal features necessary for bone regenerative medicine [29,30,31]. The main goal is to prevent CNT agglomeration and obtain optimal dispersions in a polymeric medium [32,33].

Numerous studies have shown that functionalization is the most appropriate method to improve CNT exteriors in severely acidic environments [34,35,36]. Covalent functionalization was used to create novel hydrogen bonds on the nanoparticle surfaces to obtain a certain chemical response, such as hydrogenation, oxidative stress, or alkylation. It is possible to integrate covalently functionalized CNTs into different composites to prepare carbon-based polymeric materials with increased hydrogel stability and stronger dispersibility [37,38]. They can also be used to oxidize noncovalent CNTs via π–π interactions, hydrogen bonds, and van der Waals forces to adsorb. Moreover, they bond to specific functional groups, including phenyl, hydroxyl, alkenyl, and alkyl groups. The lipophilic portion may communicate with the hydrophobic component of amphiphilic substances such as solvents, polymers, or biological particles [39,40]. Polar solvents, for example, dimethylacetamide, alcohol, and dimethylformamide, ensure a stable dispersion by stimulating greater repulsion between carbon nanoparticles. The use of surfactants and various stabilizing agents helps to form stable dispersions of CNTs.

Even though nanofibers have potential applications in tissue engineering, their toxicity cannot be disregarded. Nanomaterials can affect cells by developing reactive O_2_ species and cause apoptosis by stimulating immune reactions and chronic inflammation [41,42,43]. Previous studies have also shown that the dimensions, surface area, assemblage, process conditions, and photocatalytic process impurities impact the cytotoxicity and behavior of nanoparticles in living organisms [44,45]. Several organizations have shown that the diameter and length of nanoparticles have a substantial effect on their toxicity [46]. Depending on their size, lengthier nanotubes in tissues and organs are more likely to trigger immune responses and granuloma creation than shorter CNTs. The cytotoxicity of single-walled tubes was greater than that of multiwalled CNTs (MWCNTs), and toxic effects were higher for smaller MWCNTs than larger ones. Polyethylene glycol (PEG)–MWCNT composites exhibit biocompatibility on bone-marrow-derived stem cells of rats: PEG–MWCNT caused insignificant damage to DNA (the comet assay in Figure 2b illustrates the circular shape evenly after electrophoresis) and the dead cell rate of stem cells was low (Figure 2c) [47].

## 3. CNT–Polymer Composites for Tissue Engineering and Regenerative Medicine

### 3.1. Formation and Properties of CNT–Polymer Composites

#### 3.1.1. Natural Polymers

Because of their unusual bioactivity and high conductivity, organic biological polymers such as fibrin, chitosan, cellulose, and hyaluronic acid have been used as jaw supports and implantable devices. Nevertheless, their uncontrollable thermal degradation and low mechanical stability are indeed an ambiguity when designed to simulate the biological properties of organic matrices of bone cells. Adding CNTs to a polymer matrix increases the advantageous properties of substances by creating stable hydrogen bonds. To date, certain developments have been made in the use of carbon-based biological polymer composites for skeleton tissue engineering.

Chitosan is a polysaccharide obtained from the deacetylation of chitin. Due to its better biocompatibility, degradation, and antimicrobial activities, chitosan has shown a prominent role as a successful nanomaterial with widespread prospects [48]. Chitosan can be readily incorporated into different shapes and formulations for cellular proliferation and osteogenesis. Its water-soluble exterior helps to accumulate different negatively charged proteoglycans and facilitates post-implantation mineralization of the skeleton matrix [49,50]. CNTs aid individual chitosan molecules in becoming universally distributed in the chitosan matrix. Surface modification of chitosan and CNTs can improve the interactions between natural and inorganic phases, and high energy is required to overcome the biochemical bonding energy, which enhances the mechanical characteristics of the substrates.

**Figure 3:**
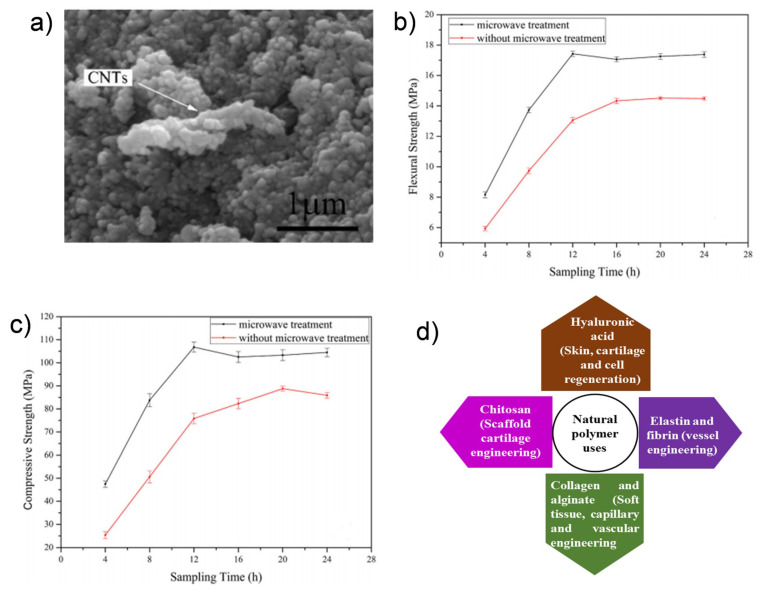
(**a**) SEM images of reduced graphene oxide (rGO), CNT and calcium phosphate cement (CPC) (RGO–CNT–CPC); (**b**) the mechanical flexural strength and (**c**) compressive strength of patterns with and without microwave treatment. Reproduced with permission from [51]; (**d**) applications of natural polymers in bone tissue regeneration.

The study showed that when 1 wt% MWCNTs was distributed uniformly across the chitosan medium, the elastic modulus and compressive strength point of the MWCNTs were distributed. Likewise, the study showed that a chitosan system mixed with just 0.8 wt% MWCNTs showed major tensile modulus and strength changes from 1.08 to 2.15 GPa and 37.7 MPa to 2.15 GPa, respectively. In other studies, it was revealed that using chitosan with CNT composites was an elegant approach for enhancing the useful material characteristics of hyaluronic acid (HA) in bone regeneration (Figure 3a–c) [51]. Figure 3d shows the natural polymers that have been used in tissue engineering and regenerative medicine.

**Figure 4:**
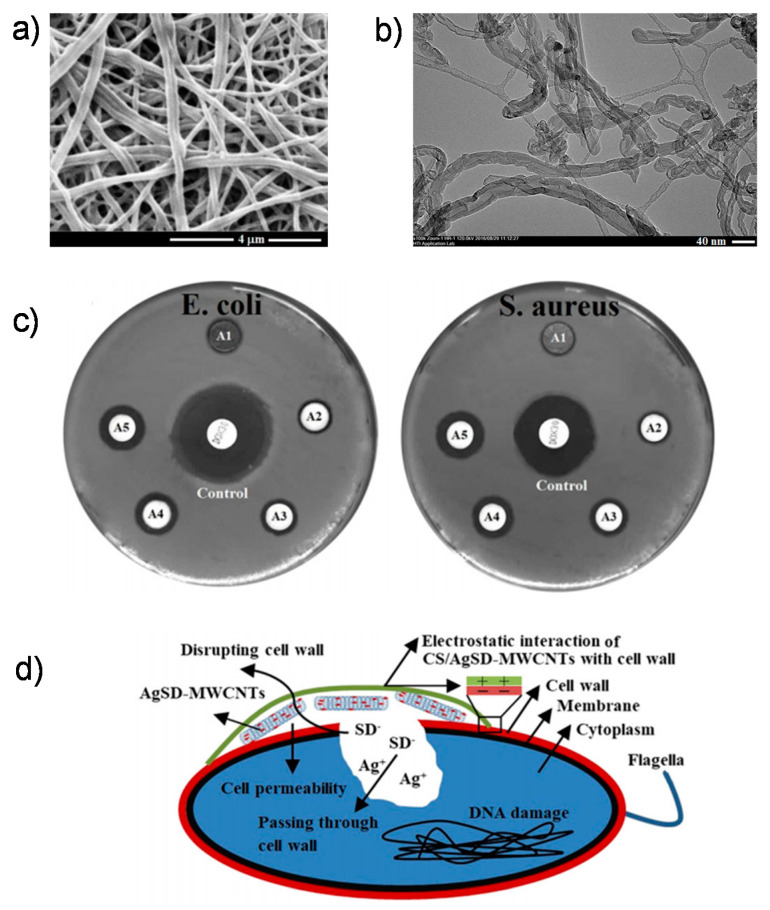
(**a**) SEM images of Mg alloys coated with chitosan–Ag sulfadiazine (CS/AgSD)–MWCNT nanofibers; (**b**) TEM images of CS/AgSD–MWCNT nanofibers; (**c**) the antibacterial activity of CS/AgSD–MWCNT nanofiber coatings for *Esherichia coli* and *Staphylococcus aureus* bacteria ((A1) is uncoated mg alloy, (A2) is CS, (A3) is CS/0.25AgSD-MWCNTs, (A4) is CS/0.5AgSD-MWCNTs, (A5) is CS/1.5AgSD-MWCNTs, and (control) is doxycycline); (**d**) the antibacterial mechanism of CS/AgSD–MWCNT nanofiber coatings. Reproduced with permission from [55].

Scientists discovered that the weight ratios of chitosan to MWCNTs affected the compressive and elastic subsystems of chitosan with multiwalled hydroxyapatite composites, expanding sharply from 33.2 to 105.5 MPa and from 509.9 to 1089.1 MPa, respectively. Since artificial mixtures cannot link organic bone in a resilient manner, chitosan–nanotube composites also have good opportunities for enhancing the distribution and configuration interplay between carbon tubes and chitosan. From a biological perspective, biocompatible chitosan–CNT composites demonstrate nontoxic effects and facilitate the differentiation of stem cells to developing skeletal cells [52]. In an in vivo experimental study of chitosan–CNT membranes inserted into rats with cranial defects, this polymer did not cause chronic inflammation over five weeks [53,54]. In another tricomposite scaffold, Ag sulfadiazine (AgSD) MWCNTs were integrated into chitosan (CS)-based nanofibers and used as a coating to enhance the antimicrobial activity of magnesium, zinc, and calcium catecholamine alloy implants for skeletal therapy (Figure 4) [55].

Collagen is the primary organic component of the skeleton, and it is essential for bone strength, hardness, and biocompatibility. Because of its bioremediation, lower antigenicity, and excellent biocompatibility, collagen has been proposed as a nanomaterial for reconstructing bone tissue [56,57,58]. Natural collagen is slightly softer and cannot be used directly as a bone substitute [59]. A mixture of CNTs can enhance the stability of collagen components, making them ideal for use in bone regeneration as biocompatible structural scaffolds. The study [60] also demonstrated that the integration of covalent bonds with workable CNTs in collagen-based frameworks is an efficient method to improve structural performance by reorganizing collagen and establishing robust heavy fiber glasses, since covalently functionalized CNTs with collagen fibers can promote the production of broader packages of fibrils. It has been shown that the use of CNTs in collagen networks facilitates bone differentiation and regeneration [61,62].

#### 3.1.2. Synthetic Polymers

Large decomposable polymers have been reported as scaffolds and implants for tissue engineering. Nevertheless, the use of these substances for artificial biomaterials is restricted in repairing bone cells due to their weak mechanical strength, low osteoinductive efficiency, and complicated applications. CNT materials have been used as reinforcing materials to incorporate their physicochemical properties with synthesized nanomaterials, to obtain ultimate composite scaffolds for bone tissue regeneration.

**Figure 5:**
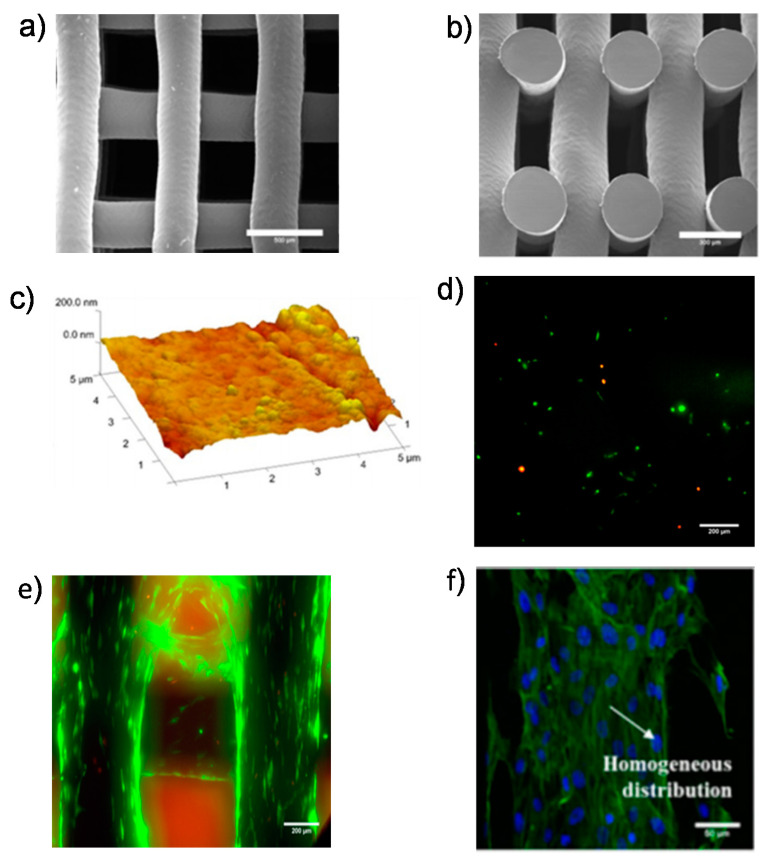
(**a**) SEM images showing the cell morphology of the polycaprolactone (PCL)–MWCNT scaffold: (**a**) top-view (scale bar: 500 µm) and (**b**) cross-section view (scale bar: 300 µm); (**c**) AFM images showing the PCL–MWCNT scaffold (scale bar: 5 µm); live/dead assay at (**d**) day 1 (scale bar: 200 µm) and (**e**) day 21 for the PCL–MWCNT scaffold (scale bar: 200 µm); (**f**) homogeneous distribution of cells on the surface of the PCL–MWCNT scaffold (scale bar: 50 µm). Reprinted with permission from [69].

Along with its excellent biocompatibility, durability, drug solubility, and ease of manufacture, polycaprolactone (PCL), a highly crystal-like polymer, is commonly used as a tissue-engineering carrier for bone tissue [63,64,65,66]. Nevertheless, it exhibits a high hydrophobicity, weak cell affinity, poor bioavailability, and inadequate load-bearing physical characteristics. Structural formulations with other components, such as CNTs, can be used to overcome these limitations [67,68]. For example, a scaffold made from PCL and MWCNTs was designed by a bend-assisted extrusion additive fabrication and provided evenly distributed pores (Figure 5) [69].

Poly(lactide-*co*-glycolide) (PLGA) is one of the most common artificial materials, even though they cannot withstand heavy components such as bone substitutes. Adding CNT to PLGA can help to overcome poor mechanical characteristics. Scientists [70] have shown that a three-dimensional PLGA membrane containing just 3% of liquid-dispersible MWCNTs had substantially improved compressive properties and a modulus that was higher than that of pure PLGA scaffolds. It also showed great cellular uptake, propagation, and mineralization.

An environmentally friendly aliphatic polyester derived from a plentiful and sustainable energy source is polylactic acid (PLA). PLA has simple molecular effectiveness, controllable deterioration, and great histocompatibility. It is a natural material used in bone engineering and bone tissue regeneration. Nonetheless, PLA’s intrinsic brittleness and low thermal stability render it incapable of withstanding large objects or stimulating cell growth. Integrating CNT into a matrix material can maximize its mechanical and surface characteristics, such as functionalization, thereby encouraging cell viability. For example, a PLA-based CNT–carboxylic acid composite was formulated using melt mixing and showed increased tensile strength and elasticity at breakage, as well as tensile strengths and thermal conductivity that were higher than that of pure PLA [71]. Likewise, a pyridine-end-functional poly-L-lactic (PLLA) system [72] effectively dispersed MWCNTs for biocompatible applications.

### 3.2. Applications

CNTs have shown good efficacy in treating infections because they act as nanocarriers of medications, genes, as receptors, as well as other delivery methods. Because of their special characteristics, CNTs have received great interest in providing new methods for treating bone infections such as osteoporosis, nonunion skeletal deficiencies, myelomatosis, and bone tumors [73,74,75].

A study by Yao et al. used CNTs and silk fibronectin to alter nano-HA scaffolds by frozen processing and cross-linking to mount dexamethasone (DEX). The application of nanotubes improved the physical and biocompatibility using NHA or PA66 scaffolds, and their carrying capacity and permeability are suitable for bone regeneration. This DEX-laden support showed an osteogenesis-stimulating outcome using stem cells, and DEX had the highest concentration of 1 mg/mL. Similar to bone marrow mesenchymal stem cells (BMSCs), CNTs exhibit a wider framework area, and a comparatively higher drug loading can be achieved if medications are immobilized into pipe holes or fixed to the layers [76].

Researchers have made NPs based on chitosan–CNTs for administering low doses of isoniazid to manage bacterial ulcers. Wet lab analysis revealed that NPs from chitosan–CNTs substantially expanded the release time to seven days and stabilized the isoniazid release profile while maintaining isoniazid’s biological role. The mouse prototype of a tubercular ulcer revealed that nanoparticles transported isoniazid to the ulcer site and killed *Mycobacterium tuberculosis*. Such results demonstrated their ability to lower the cytotoxicity of isoniazid and increase its sensitivity. Such carbon-tube-based NCs may be overloaded with anticarcinogenic medicine. Thus, the production of isoniazid-loaded chitosan–CNT (INH–CS–CNT) nanoparticles is a novel approach for treating skeletal deficiencies and secondary injuries [77].

The sustained release of a medication directly affects its efficiency. The use of CNTs as a medicine delivery system can overcome existing drug discovery drawbacks, such as low drug solubility, fast inactivation, and decreased bioactivity. Research has shown that CNT-containing composites have strong sustained release characteristics and extended optimum release [78]. Costantini et al. suggested replacing bone with nanotubes, chitosan, and hydroxyapatite to monitor the exposure of divergent standard drugs such as ibuprofen, isothiocyanate–dextran fluorescein, and ibuprofen sodium. In conjunction with chitosan, the introduction of CNTs minimized the maximum leakage of organic compounds, ibuprofen, and ibuprofen sodium for 48 h. Upon introduction, the drug relief of the water-soluble molecule fluorescein isothiocyanate-based dextran was smaller than those of others. The findings demonstrated that CNTs can regulate the release of hydrophilic drugs with both higher and lower molecular masses, which is a valuable multidisciplinary drug discovery platform for skeletal tissue engineering [79].

Lu et al. designed superparamagnetic-like CNT hydroxyapatite composite scaffolds. The fluid-like structure displayed excellent mechanical strength and an ideal pore diameter for osteoconduction and bone formation, with enlarged particles of 1 to 2 mm and tiny pores of 20 to 300 µm. Interestingly, these porous CNT–hydroxyapatite scaffolds displayed superparamagnetic behavior, with an emu/gram saturation magnetization that was beneficial for scaffolds to recruit and accumulate stem cells or certain biologically active molecules in vivo as a development factor [80]. Table 1 provides examples of the diverse applications of CNTs in skeletal tissues.

## 4. Challenges and Prospects

Although CNTs have a promising future in tissue engineering uses for enhancing biochemical, mechanical, and electrical characteristics, they also display several shortcomings that restrict their medical applications. For instance, there is a strong need for technologies and techniques to assess and evaluate the toxicity, carcinogenic effects, and teratogenic effects of CNTs. Secondly, the toxicity, carcinogenic, and teratogenic effects of CNTs are both extremely dose-dependent and exposure-dependent. CNTs are often utilized in nominal amounts, so they are labeled nontoxic. The biological accumulation of CNTs is well-established. Consequently, any CNTs utilized in the body can be absorbed and produce side effects in organelles or tumors, or negative effects in reproductive organs and infants. Furthermore, although various devices use CNTs, there have been some theoretical and practical holes in the understanding of the various hazards of this nanomaterial. Currently, nano-specific threat assessments, involving relevant data criteria and research methods, do not yet have global standards, and CNT risk assessments are tedious and expensive. Companies are generally dedicated to evaluating the protection of CNT-based products and enforcing the required safety precautions. The regulatory instruments are not nano-specific. For instance, data specifications for chemical warnings, classification features, and health data paper marking standards are not yet commonly available; therefore, preventative measures are required before implementing CNTs, which can potentially lead to biological accumulation.

## 5. Conclusions

CNTs display outstanding bioactivity and very well-established chemical surface techniques and will be extremely useful in many biological devices. CNTs have also been used to improve the electric pairing among decellularized molecules and increase the proliferation rate of skin cells. The accuracy of CNTs has also been explored with incredible potential regarding antimicrobial growth. CNTs are beginning to emerge as outstanding materials that can provide new ideas and prospects for the future for the rejuvenation and manufacture of bone cells. Nevertheless, to transition from exploratory findings to clinical procedures, many difficulties need to be overcome. Prospective regenerative medicine solutions may involve multiple elements to provide complete control over the assimilation, tracking, and long-term stability of tissue engineering of these elements. Nanoscale drug delivery aims to develop progressive delivery systems and assess their systemic cytotoxic properties and immune responses. Such research can best explain the biological compatibility of many nanomaterial delivery methods, which can guide future research in a cost-effective approach with a higher success rate. To this end, the distinct types of nanoparticles of various materials include a powerful toolset for tissue engineering synthetic tissue functionalization.

## Figures and Tables

**Table 1:** CNT applications in bone tissue engineering.

No.	Materials Used	Uses of CNTs	Significance	Ref.
**Natural Polymers**				
1	Chitosan	Nanocomposite films and jaw skin scaffolds	Enhanced biologically active characteristics, tensile strength, and cell proliferation	[48,81,82]
2	Collagen	Three-dimensional CNT covered jaw and jaw repair biological materials	Improved functionality and mechanical stability	[60,83]
3	Microbial cellulose	Bone tissue scaffolds	Enhanced mechanical characteristics and proliferation	[84]
4	Silk fibroin	Nanocomposite films	Supports jaw cell adhesion and development	[85]
5	Collagen–hydroxyapatite and gelatin–chitosan	Jaw scaffold materials	Enhanced stiffness, elastic modulus, elongation rate, and cell viability	[86,87]
**Calcium Phosphate**				
1	Hydroxyapatite	Jaw implant materials	Enhanced jaw integration, mechanical features, and novel bone materialization	[88,89,90]
2	Calcium phosphate	Injectable jaw substitutes	Enhanced compressive strength and hydroxyapatite (HA) crystal formation	[51]
3	β-tricalcium phosphate	Jaw repair materials	Enhanced HA and apatite formation	[91]
**Synthetic Polymers**				
1	Polylactic acid	NC materials and jaw tissue engineering	Enhanced tensile strength and thermal solidity and possesses electrical conductivity	[71,92]
2	Poly(lactide-*co*-glycolide)	Jaw repair and tissue scaffolds	Exhibits better tissue and cell compatibility, enhanced mechanical strength and proliferation	[15,70,93]
3	Polycaprolactone	Three-dimensional jaw scaffolds	Enhanced cell proliferation and tensile strength	[69,94]
